# Association of habitually low intake of dietary selenium with new-onset stroke: A retrospective cohort study (2004–2015 China Health and Nutrition Survey)

**DOI:** 10.3389/fpubh.2022.1115908

**Published:** 2023-01-09

**Authors:** Huanxiang Zhang, Hongbin Qiu, Shanjie Wang, Yiying Zhang

**Affiliations:** ^1^Department of Epidemiology and Biostatistics, School of Public Health, Jiamusi University, Jiamusi, China; ^2^Department of Cardiology, The Second Affiliated Hospital of Harbin Medical University, Harbin, China

**Keywords:** dietary selenium, stroke, CHNS, cohort study, adults

## Abstract

**Background:**

As an essential trace element in the body, selenium is associated with the development of many diseases. The purpose of this study was to explore the association between dietary selenium intake and new-onset stroke risk in Chinese adults.

**Methods:**

Adults aged ≥18 years in the China Health and Nutrition Survey (CHNS) from 2004 to 2015 were enrolled. Participants were divided into five groups according to the quintile of dietary selenium intake: Q1 (≤ 29.80 μg/day), Q2 (29.80–38.53 μg/day), Q3 (38.53–47.23 μg/day), Q4 (47.23–60.38 μg/day), Q 5(>60.38 μg/day). Cox proportional-hazards model was used to explore the effect of dietary selenium on new-onset stroke. Restricted cubic spline (RCS) was used to visualize the dose-response relationship between dietary selenium and the risk of morbidity.

**Results:**

A total of 11,532 subjects were included, and 271 (2.35%) of them developed stroke during a mean follow-up of 6.78 person-years. Compared with the lowest selenium intake group, the *HR* and 95%*CI* of stroke in the participants with selenium intake of Q2, Q3, Q4 and Q5 were: 0.85 (0.59, 1.21), 0.62 (0.42, 0.92), 0.43 (0.28, 0.68), 0.49 (0.30, 0.82), respectively. There was an L-shaped relationship between dietary selenium and stroke (nonlinear *P*-value = 0.0420). The *HR* and 95%*CI* of developing stroke was 0.75 (0.65, 0.87) in participants with selenium intake ≤ 60 μg/day.

**Conclusions:**

The L-shaped negative association between dietary selenium and stroke in Chinese adults which indicated that dietary selenium should be improved to a certain level to prevent stroke.

## 1. Introduction

Stroke is one of the most common cardiovascular diseases. Globally, stroke remains the second leading cause of death and the third leading cause of disability. In addition, the global burden of disease (GBD) showed that in 2019, age-standardized stroke-related death rates were 3.6 times higher in the World Bank's low-income group than in the World Bank's high-income group, and age-standardized stroke-related DALY rates were 3.7 times higher in the low-income group than in the high-income group (1). In China, the disease burden of stroke remains significant despite declines in both age-standardized incidence and mortality of stroke from 1990 to 2019 (the incidence of stroke increased by 86.0% in 2019 compared to 1990, with 3.94 million new cases) (2). While the burden of stroke can be reduced through modifiable risk factors such as behavior, air pollution and diet, traditional risk factors do not account for all stroke risk (3). Therefore, new modifiable factors need to be identified. In recent years, more and more attention has been paid to the role of trace elements in cardiovascular diseases.

As an essential element for human body, selenium is mainly involved in a variety of metabolic processes, including regulating thyroid hormone (4), oxidative stress response (5), antioxidant mechanism (6) and immune response (7–9) through 25 selenocysteine, as the active center of selenium protein (10). As a trace element, the health effects of selenium exposure levels are controversial. Some studies have shown adverse health effects from high levels of selenium exposure. High levels of selenium exposure (including plasma selenium, dietary selenium, and environmental selenium) can significantly increase the risk of cardiovascular disease, diabetes, dyslipidemia, and other diseases (11–15). However, other studies have found that increasing selenium levels can be beneficial. Clinical randomized trials and several large national surveys have shown that increased selenium intake or circulating concentrations of selenium are associated with reduced risk of cancer mortality, all-cause mortality, and stroke (16–21). Different from the above results, Jenkins and Ingles et al. showed that the use of nutrient supplements did not bring significant benefits to cardiovascular diseases (22, 23). Outzen et al. found that neither toenail selenium nor plasma selenium was associated with advanced prostate cancer (24).No significant association was found between selenium intake and metabolic syndrome and its parameters (25). The different results of these studies may be attributed to the different forms of selenium supplementation (26, 27), the different levels of selenium exposure (sufficient or not) (25, 28), the different evaluation indicators of selenium (28).

Inadequate (29) or excessive (11) of selenium intake may be associated with a number of adverse health outcomes. Selenium intake varies greatly in different geographical locations (30) and dietary habits (31). Combs Jr said that although it is difficult to quantify the total number of people in the world who are selenium deficient, about 500 million to 1 billion people are selenium deficient (31). Selenium intake is higher in western countries such as the United States, with an average daily intake of more than 111 μg (32). European and Middle Eastern countries have suboptimal selenium intake and status (33). China is a country of selenium deficiency, with an average selenium intake of 41.1 μg/day according to national surveys (34). It is estimated that 39–61% of Chinese residents' daily selenium intake is lower than the WHO/FAO recommendation (26–34 μg/day) (35), and the health problems caused by selenium deficiency in China needed special attention. Previous studies on dietary selenium and stroke were mostly limited to western countries with relatively adequate selenium intake. The conclusion may not be suitable for Chinese people with habitual low selenium intake.

Therefore, our study aimed to explore the relationship between dietary selenium intake and new-onset stroke in the general population of China, using a retrospective cohort study method.

## 2. Materials and methods

### 2.1. Study population

**Figure 1:**
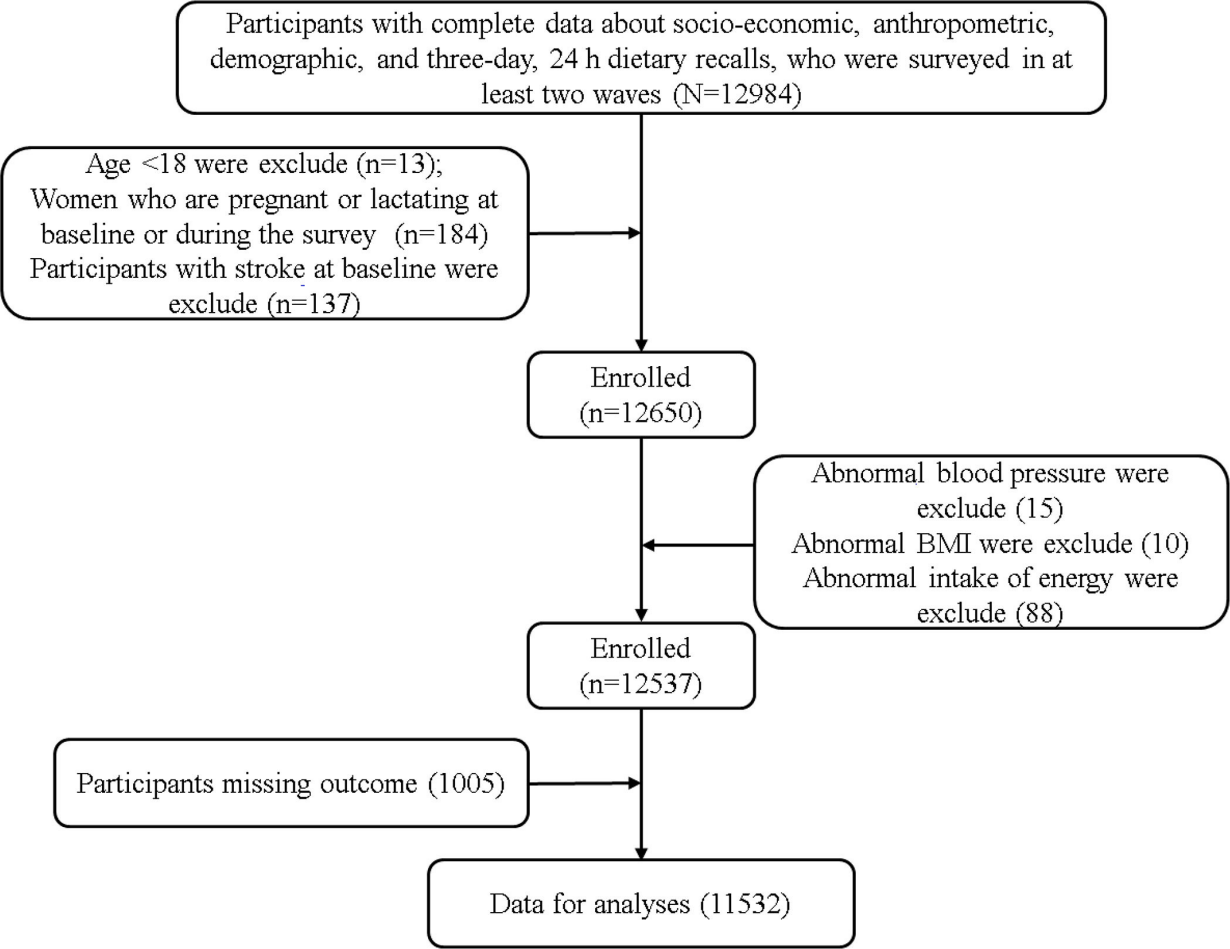
Flow chart for inclusion and exclusion of research subjects.

The data of this study comes from CHNS (China Health and Nutrition Survey), a longitudinal follow-up survey based on community population. The survey included socio-economic status, health services and nutritional and dietary status. Please refer to relevant literature or official website (http://www.cpc.unc.edu/projects/china.) for specific survey population and sampling methods (36). Since CHNS has provided more accurate dietary data after 2004 and can be obtained on the official website, the subjects of this study were adults aged ≥18 years who participated in the survey from 2004 to 2015. Participants who have received at least two rounds, and have complete sociodemographic indicators, socioeconomic indicators, lifestyle, anthropometric indicators, physical health status indicators and three consecutive 24-h diet recall data were considered as valid subjects. The exclusion criteria include: (1) Age <18, (2) Pregnant or lactating women, (3) Participants with stroke at baseline, (4) Exceeding the energy intake limit (Male: >6,000 kcal or <800 kcal; Female: >4,000 or <600 kcal) (37), systolic blood pressure (SBP) <40 mmHg or >300 mmHg, diastolic blood pressure (DBP) <30 mmHg or >200 mmHg, body mass index (BMI) <14 kg/ m^2^ or >45kg/ m^2^ or other unreliable observations. Finally, 11,532 participants were included in the final analysis (Figure 1). All individuals had signed a written informed consent prior to participating in the study, which was in accordance with the Declaration of Helsinki and approved by the institutional review committees of the University of North Carolina at Chapel Hill and the National Institute of Nutrition and Food Safety, Chinese Center for Disease Control and Prevention (No.201524-1) (38).

### 2.2. Assessments of dietary selenium intake

Three consecutive 24-h dietary recalls were used to assess dietary intake of participating individuals and household food consumption (including the consumption of edible oil and condiments) over the same 3-day period was calculated by subtracting the ending inventory from the starting inventory. The accuracy of dietary data can be ensured by comparing the average daily dietary intake of households calculated from household surveys with the average daily dietary intake of individuals calculated from their 24-h recall data. Food consumption data were converted into the dietary selenium intake using Chinese Food Composition Tables (FCTs). The cumulative average dietary intake from baseline to the latest year before the year of the first stroke event or the end of follow-up was used for analysis, because it can better reflect long-term dietary intake and minimize within-person variation.

### 2.3. Outcome identification

The outcome variable was whether stroke occurred during the follow-up period. The on-site investigators asked the participants whether they had stroke through interviews: “Has a doctor from a public hospital at or above the county level ever given you a diagnosis of stroke (no, yes, unknown). New-onset stroke was defined as a self-reported physician diagnosis of stroke during follow-up and a certificate of stroke diagnosis should be provided. For participants who provided inconsistent answers during the follow-up, the first recorded stroke event was adopted to limit recall bias. The follow-up time was the date of the first discovery of stroke.

### 2.4. Assessments of covariates

The demographic, socioeconomic and lifestyle information of the participants was obtained through face-to-face interviews conducted by trained field investigators using well-designed questionnaires, including age, sex, race, site, marital status, education level, activity level, smoking status, alcohol intake. Body mass index (BMI) was calculated by dividing body weight by the square of height (kg/m^2^). Weight and height were obtained by measuring to an accuracy of 0.1 kg in weight and 0.1 cm in height, after removing shoes and heavier clothing. Hypertension was defined as SBP ≥140 mmHg, DBP ≥90 mmHg, or taking antihypertensive medication according to the guidelines for preventing and treating hypertension in China (2010), or providing a self-reported diagnosis by a physician in public hospitals above the county level. In a quiet environment, the participants sat quietly for more than 5 min. Then, after removing their heavy clothing, their blood pressure was measured three times by a trained health nurse using a calibrated mercury sphygmomanometer, and the average of the three blood pressure measurements was used for the final analysis. Diabetes was defined based on self-report of diagnosis by a physician in public hospitals above the county level, or using oral medicine, injection of insulin to control blood glucose. Myocardial infarction was defined based on self-report of diagnosis by a physician in public hospitals above the county level. The intake of energy, dietary fiber, niacin, vitamin C, vitamin E, calcium, iron, zinc, magnesium, copper and manganese is calculated in the same way as that of dietary selenium.

### 2.5. Statistical analysis

SAS version 9.2 (SAS Institute, Cary, North Carolina, USA) and R version 4.0.5 were used for statistical analysis. Participants were categorized into quintile groups: Q1(≤ 29.80 μg/day), Q2(29.80–38.53 μg/day), Q3(38.53–47.23 μg/day), Q4(47.23–60.38 μg/day), Q5(>60.38 μg/day). According to their selenium intake levels and Q1 was considered as exposure group. Continuous variables that did not meet the normal distribution were represented by the median and interquartile range, while categorical values were expressed as numbers (percentage). Differences between groups were evaluated by using Kruskal-Wallis H test, and the chi-square test for the categorical variables.

Restricted cubic spline functions, with 3 knots (10, 50, 90%), combined with Cox proportional hazards models were used to visualize the relationship between dietary selenium intake and the risk of developing stroke, after adjusting the variables of model 4. Then, anova function was used to test whether the above relationship was linear. When non-linearity was detected, a two-segment linear model was established on both sides of the inflection point with the recommended nutrient intake (RNI) of selenium: 60 μg/day as the inflection point. Previous studies have observed associations between nutritional intake and health outcomes influenced by age, sex, BMI, site, smoking status, alcohol consumption, hypertension. Therefore, stratified analyses assessed whether these factors altered the association. The test level was α = 0.05, two-tailed.

Four Cox proportional hazards regression models were used to calculate the hazard rations (*HR*s) and 95% confidence intervals (*CI*) of new-onset stroke. Model 1 was non-adjusted; Model 2 was adjusted for age, gender, race and energy intake. Model 3 was additionally adjusted for site, marital status, education and activity level, smoking and drinking status, body mass index, hypertension, diabetes and myocardial infarction based on model 2; Model 4 was further adjusted for intake of dietary fiber, niacin, vitamin C, vitamin E, calcium, iron, zinc, magnesium, copper, manganese based on model 3.

## 3. Results

### 3.1. Information of dietary selenium intake

A total of 11,532 adults subjects were eventually enrolled in this study, which consisted of 5,389 males and 6,143 females. Among the entire participants, 79.59% had selenium intake lower than RNI (60 μg/day for aged 18 years and above), 65.05% had selenium intake lower than estimated average requirement (EAR, 50 μg/day for aged 18 years and above), and the mean intake of selenium was 48.01 (standard deviation = 14.44) μg/day (39). More details of selenium intake were shown in Table 1.

**Table 1:** Selenium intake among Chinese adults.

**-**	**Total**	**Male**	**Female**
Subjects (*n*)	11,532	5,389	6,143
Dietary Se intake (μg/d)	47.36 ± 28.99	51.53 ± 33.53	43.71 ± 23.73
Lower than RNI, no. (%)	9,178(79.59)	3,947(73.24)	5,231(85.15)
Lower than EAR, no. (%)	7,502(65.05)	3,071(56.99)	4,431(72.13)

Mean ± SD for continuous variable and numbers (percentage) for categorical variables. RNI, Recommended Nutrient Intake (60 μg/day for aged 18 years and above); EAR, Estimated Average Requirement (50 μg/day for aged 18 years and above).

### 3.2. Basic characteristics

The baseline characteristics of the study population according to the intake of selenium were shown in Table 2. Participants with higher intake of dietary selenium were more likely to be younger, male, Han nationality, live in urban site, never married, ≥middle school education levels, do light or moderate physically activity, smokers and drinkers, had a higher intake of energy, dietary fiber, niacin, vitamin C, vitamin E, calcium, iron, zinc, magnesium, copper, manganese, and less likely to be divorced, separated or widowed, ≤ primary school education levels, do heavy physically activity, hypertension, and stroke. The result of comparing the indicators between non-stroke and new-onset stroke was shown in Supplementary Table S1.

**Table 2:** Baseline characteristics of participants according to quintiles of dietary selenium intake.

**Characteristics**	**Dietary selenium intake (**μ**g/day)**					***P*-value**
	**Q1 (≤ 29.80)**	**Q2 (29.80–38.53)**	**Q3 (38.53–47.23)**	**Q4 (47.23–60.38)**	**Q5 (> 60.38)**	
N	2,306	2,306	2,306	2,307	2,307	
Male, no. (%)	793 (34.39)	946 (41.02)	1,044 (45.27)	1,189 (51.54)	1,417 (61.42)	<0.0001
Han race, no. (%)	1,915 (83.04)	1,938 (84.04)	2,087 (90.50)	2,146 (93.02)	2,190 (94.93)	<0.0001
Urban, no. (%)	757 (32.83)	782 (33.91)	851 (36.90)	931 (40.36)	1,181 (51.19)	<0.0001
Smoking, no. (%)	589 (25.54)	665 (28.84)	699 (30.31)	796 (34.50)	924 (40.05)	<0.0001
Drinking, no. (%)	535 (23.20)	678 (29.40)	716 (31.05)	872 (37.80)	1,037 (44.95)	<0.0001
Hypertension, no. (%)	650 (28.19)	534 (23.16)	578 (25.07)	557 (24.14)	590 (25.57)	0.0015
Diabetes, no. (%)	61 (2.65)	36 (1.56)	57 (2.47)	48 (2.08)	62 (2.69)	0.0563
Myocardial infarction, no. (%)	16 (0.69)	9 (0.39)	8 (0.35)	10 (0.43)	5 (0.22)	0.1455
Stroke, no. (%)	70 (3.04)	63 (2.73)	55 (2.39)	40 (1.73)	43 (1.86)	0.0146
**Marital status, no. (%)**						
Never married	135 (5.85)	126 (5.46)	130 (5.64)	130 (5.64)	167 (7.24)	<0.0001
Married	1,852 (80.31)	1,966 (85.26)	2,017 (87.47)	2,075 (89.94)	2,030 (87.99)	
Divorced, separated, widowed, etc	319 (13.83)	214 (9.28)	159 (6.90)	102 (4.42)	110 (4.77)	
**Education level, no. (%)**						
≤ Primary school	1,244 (53.95)	999 (43.32)	874 (37.90)	749 (32.47)	533 (23.10)	<0.0001
Middle school	614 (26.63)	750 (32.52)	775 (33.61)	789 (34.20)	808 (35.02)	
≥High school	448 (19.43)	557 (24.15)	657 (28.49)	769 (33.33)	966 (41.87)	
**Activity level, no. (%)**						
Light	1,151 (49.91)	1,135 (49.22)	1,205 (52.25)	1,264 (54.79)	1,446 (62.68)	<0.0001
Moderate	309 (13.40)	347 (15.05)	348 (15.09)	355 (15.39)	393 (17.04)	
Heavy	846 (36.69)	824 (35.73)	753 (32.65)	688 (29.82)	468 (20.29)	
Age (years)	52.00 (39.00, 64.00)	48.00 (37.00, 59.00)	47.00 (37.00, 58.00)	47.00 (37.00, 55.00)	47.00 (36.00, 56.00)	<0.0001
BMI (kg/m^2^)	22.43 (20.44, 24.95)	22.59 (20.55, 25.07)	22.89 (20.88, 25.48)	23.44 (21.33, 25.71)	23.61 (21.47, 25.95)	<0.0001
Energy (kcal/day)	1,330.18 (1,054.27, 1,623.00)	1,654.24 (1,387.39, 1,918.67)	1,788.58 (1,536.23, 2,095.50)	1,953.19 (1,673.74, 2,273.97)	2,148.03 (1,823.86, 2,567.34)	<0.0001
Dietary fiber (g/day)	8.59 (6.45, 11.10)	10.11 (7.83, 12.97)	11.26 (8.74, 14.39)	12.51 (9.50, 16.27)	13.99 (10.33, 18.70)	<0.0001
Niacin (mg/day)	10.87 (8.61, 13.02)	13.37 (10.96, 15.71)	15.07 (11.85, 17.72)	16.22 (12.41, 19.74)	18.73 (14.33, 23.57)	<0.0001
Vitamin C (mg/day)	69.60 (46.47, 98.70)	80.83 (57.37, 107.56)	86.67 (62.95, 113.50)	89.47 (65.05, 119.39)	98.19 (68.68, 131.80)	<0.0001
Vitamin E (mg/day)	6.90 (4.81, 9.82)	9 (6.68, 12.34)	10.67 (7.88, 14.13)	12.27 (9.19, 16.47)	15.09 (11.31, 20.13)	<0.0001
Calcium (mg/day)	265.54 (198.90, 354.99)	331.21 (263.57, 412.63)	366.77 (297.00, 460.13)	415.39 (326.47, 516.26)	523.80 (400.88, 678.5)	<0.0001
Iron (mg/day)	14.18 (11.30, 17.18)	17.17 (14.29, 20.36)	19.13 (15.96, 22.78)	20.91 (17.69, 25.47)	24.26 (19.94, 30.33)	<0.0001
Zinc (mg/day)	7.90 (6.35, 9.43)	9.68 (8.07, 11.38)	10.69 (8.75, 12.56)	11.63 (9.49, 13.70)	13.37 (11.07, 16.21)	<0.0001
Magnesium (mg/day)	214.31 (170.33, 263.15)	255.30 (213.32, 302.74)	277.58 (238.87, 327.76)	308.40 (262.62, 365.48)	350.19 (298.87, 417.30)	<0.0001
Copper (mg/day)	1.41 (1.05, 1.81)	1.69 (1.30, 2.07)	1.83 (1.46, 2.28)	1.99 (1.60, 2.49)	2.31 (1.84, 3.08)	<0.0001
Manganese (mg/day)	4.53 (3.30, 5.76)	5.26 (4.08, 6.55)	5.52 (4.39, 6.96)	5.82 (4.65, 7.37)	6.11 (4.82, 7.75)	<0.0001

Dietary selenium intake (μg/day).

Median (q1, q3) for continuous variables and numbers (percentage) for categorical variables. BMI: body mass index.

### 3.3. Correlation between dietary selenium intake and risk of developing stroke

There was no covariance between the independent variables (Supplementary Table S2). 271 participants developed stroke after 78,178 person-years of follow-up.

**Figure 2:**
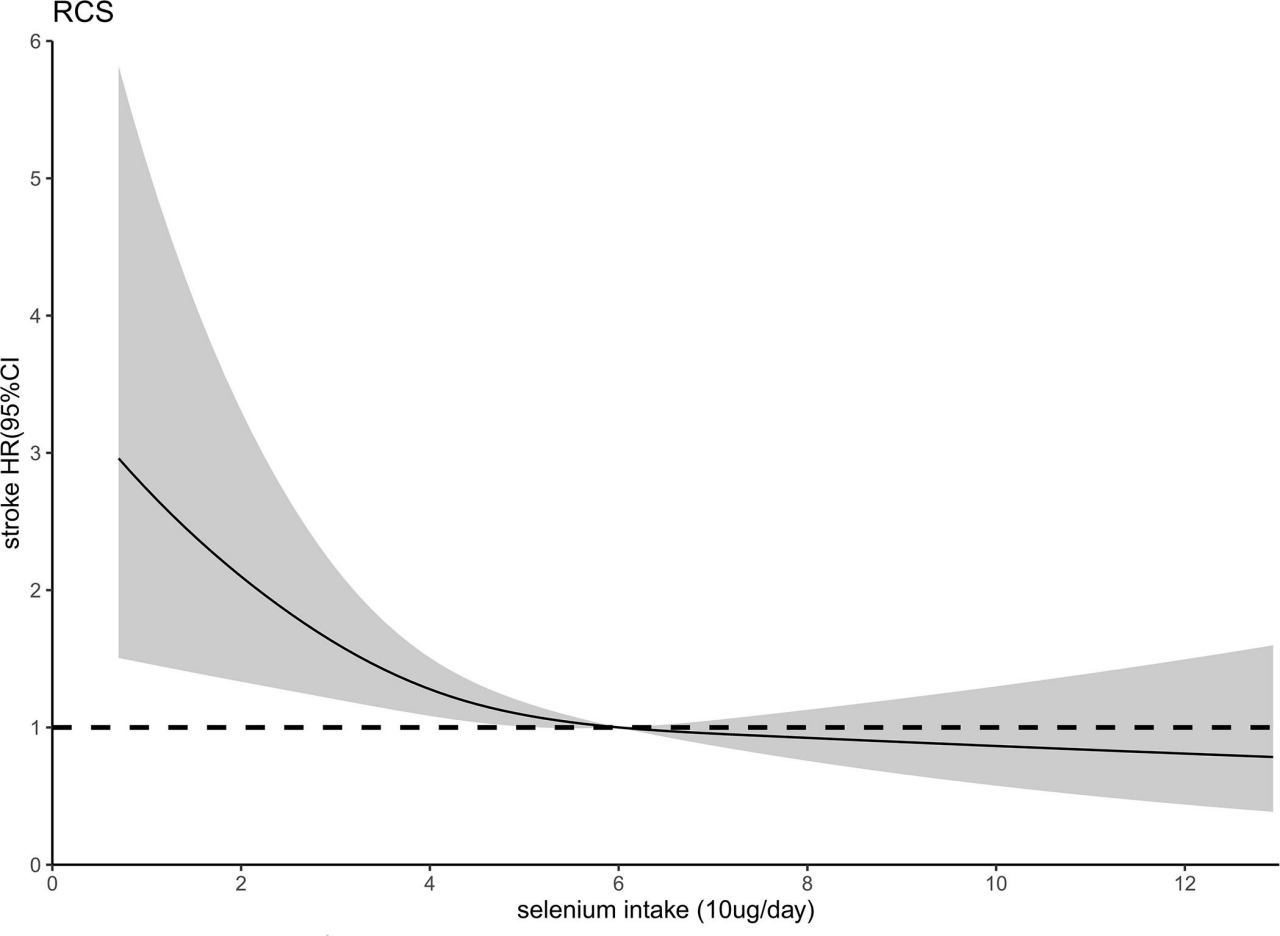
Relation of selenium intake with risk of developing stroke. Adjusted for age, gender, race, energy intake, site, marital status, education level, activity level, smoking status, drinking status, BMI, hypertensions, myocardial infarction, diabetes, dietary fiber, niacin, vitamin C, vitamin E, calcium, iron, zinc, magnesium, copper, and manganese.

Fully adjusted Cox proportional hazards regression model combined with restricted cubic spline function was conducted to clarify the dose-response relationship between dietary selenium intake and the risk of new-onset stroke, and an L-shaped (*P*-value for nonlinearity was 0.0420) association was found (Figure 2). A linear model and a two-stage linear model, with RNI for selenium (60 μg/day) as the cut-off point, were used to evaluate the relationship between dietary selenium intake and the risk of new-onset stroke, respectively (Table 3). When dietary selenium ≤ 60 μg/day, the risk of stroke significantly decreased with the increase of dietary selenium, *HR* and 95%*CI* was 0.95(0.65, 0.87). However, this significance disappeared when selenium >60 ug/day, *HR* and 95%*CI* were 0.94(0.81, 1.08).

**Table 3:** Threshold effect of dietary selenium intake on risk of developing stroke.

**Dietary Se intake (μg/day)**	**Model 1**	**Model 2**	**Model 3**	**Model 4**
**Linear model**				
*HR* (95%*CI*)	0.90 (0.84, 0.97)	0.91 (0.84, 0.99)	0.88 (0.81, 0.96)	0.88 (0.80, 0.96)
*P*-value	0.003	0.0215	0.0055	0.0065
**2-piecewise linear model**				
*HR^a^* (95%*CI*), Se ≤ 60	0.79 (0.70, 0.88)	0.82 (0.72, 0.94)	0.76 (0.65, 0.88)	0.75 (0.65, 0.87)
*P*-value	<0.0001	0.005	0.0003	0.0002
*HR^b^* (95%*CI*), Se > 60	0.98 (0.87, 1.11)	0.97 (0.87, 1.09)	0.95 (0.83, 1.09)	0.94 (0.81, 1.08)
*P*-value	0.7731	0.6226	0.465	0.3668
*P*-value for non-linearity	<0.0001	0.0400	0.0243	0.0420

HR, hazard ratio, CI, confidence intervals. ^a^Risk of stroke when dietary selenium ≤ 60 μg/day, ^b^Risk of stroke when dietary selenium ≤ 60 μg/day. Model 1: non-adjusted. Model 2: adjusted for age, gender, race, and energy. Model 3: adjusted for age, gender, race, energy, site, marital status, education level, activity level, smoking status, drinking status, BMI, hypertension, diabetes, and myocardial infarction. Model 4: adjusted for age, gender, race, energy intake, site, marital status, education level, activity level, smoking status, drinking status, BMI, hypertension, diabetes, myocardial infarction, dietary fiber, niacin, vitamin C, vitamin E, calcium, iron, zinc, magnesium, copper, and manganese.

Table 4 showed that selenium intake has a negative impact on the risk of stroke in the four Cox proportional hazards models. After controlling for age, gender, race, site, marital status, education level, activity level, smoking status, drinking status, hypertension, diabetes, myocardial infarction, BMI, and the intake of energy, dietary fiber, niacin, vitamin C, vitamin E, calcium, iron, zinc, magnesium, copper, manganese, we found that the adjusted *HR*s and 95% (*CI*) of stroke in Q2 to Q5 of selenium intake were 0.85 (0.59, 1.21), 0.62 (0.42, 0.92), 0.43 (0.28, 0.68), 0.49 (0.30, 0.82), respectively, compared with Q1, and the trend *P*-value of quintile was 0.0029. Furthermore, compared to Q4 of selenium intake (contains RNI and EAR of selenium), the risk of developing stroke was increased by 2.31 times in those consuming ≤ 29.80 μg selenium daily (*HR* = 2.31, 95% *CI*: 1.48–3.61) and by 1.95 times (*HR* = 1.95, 95% *CI*: 1.29–2.97) in those consuming 29.80 to 38.53 μg daily.

**Table 4:** Hazard ratios for stroke according to quintiles of dietary selenium intake.

	**Dietary selenium intake (**μ**g/day)**					***P*-value**
**Total population**	**Q1 (** ≤ **29.80)**	**Q2 (29.80–38.53)**	**Q3 (38.53–47.23)**	**Q4 (47.23–60.38)**	**Q5 (**> **60.38)**	
Cases	70	63	55	40	43	–
N	2,306	2,306	2,306	2,307	2,307	–
Person-years	13,466	16,580	17,119	16,631	14,382	–
Incidence density	5.20	3.80	3.21	2.41	2.99	–
Incidence rate (%)	3.04	2.73	2.39	1.73	1.86	–
Model 1 *HR* (95%*CI*)^a^	1.00	0.73 (0.52, 1.03)	0.62 (0.43, 0.88)	0.46 (0.31, 0.68)	0.58 (0.40, 0.85)	0.0011
Model 2 *HR* (95%*CI*)^a^	1.00	0.85 (0.60, 1.21)	0.69 (0.47, 1.01)	0.51 (0.33, 0.79)	0.58 (0.37, 0.91)	0.0242
Model 3 *HR* (95%*CI*)^a^	1.00	0.82 (0.58, 1.17)	0.61 (0.41, 0.90)	0.44 (0.28, 0.68)	0.49 (0.30, 0.79)	0.0028
Model 4 *HR* (95%*CI*)^a^	1.00	0.85 (0.59, 1.21)	0.62 (0.42, 0.92)	0.43 (0.28, 0.68)	0.49 (0.30, 0.82)	0.0029
Model 1 *HR* (95%*CI*)^b^	2.17 (1.47, 3.20)	1.58 (1.06, 2.35)	1.34 (0.89, 2.10)	1.00	1.25 (0.81, 1.93)	0.0011
Model 2 *HR* (95%*CI*)^b^	1.95 (1.23, 3.00)	1.66 (1.10, 2.50)	1.35 (0.90, 2.03)	1.00	1.12 (0.73, 1.74)	0.0242
Model 3 *HR* (95%*CI*)^b^	2.29 (1.47, 3.57)	1.88 (1.24, 2.85)	1.39 (0.92, 2.10)	1.00	1.11 (0.71, 1.73)	0.0028
Model 4 *HR* (95%*CI*)^b^	2.31 (1.48, 3.61)	1.95 (1.29, 2.97)	1.43 (0.95, 2.17)	1.00	1.14 (0.72, 1.79)	0.0029

Incidence density, 1/1,000 person-years; HR, hazard ratio; CI: confidence intervals. ^a^The lowest quintile (Q1) was regarded as the reference, ^b^The fourth quintile (Q4) was regarded as the reference. Model 1: non-adjusted. Model 2: adjusted for age, gender, race, and energy. Model 3: adjusted for age, gender, race, energy, site, marital status, education level, activity level, smoking status, drinking status, BMI, hypertension, diabetes, and myocardial infarction. Model 4: adjusted for age, gender, race, energy intake, site, marital status, education level, activity level, smoking status, drinking status, BMI, hypertension, diabetes, myocardial infarction, dietary fiber, niacin, vitamin C, vitamin E, calcium, iron, zinc, magnesium, copper, and manganese.

To test the robustness of the association, we further performed a sensitivity analysis. Firstly, dietary selenium intake was divided into 3 groups according to RNI and EAR, and the risk of stroke was lowest in the group with selenium intake between EAR to RNI, and the results have not changed substantially (Supplementary Table S3). Secondly, after imputation for missing values by multiple imputation, the associations remained consistent with before results (Supplementary Table S4).

### 3.5. Stratified correlations between dietary selenium intake and stroke

**Figure 3:**
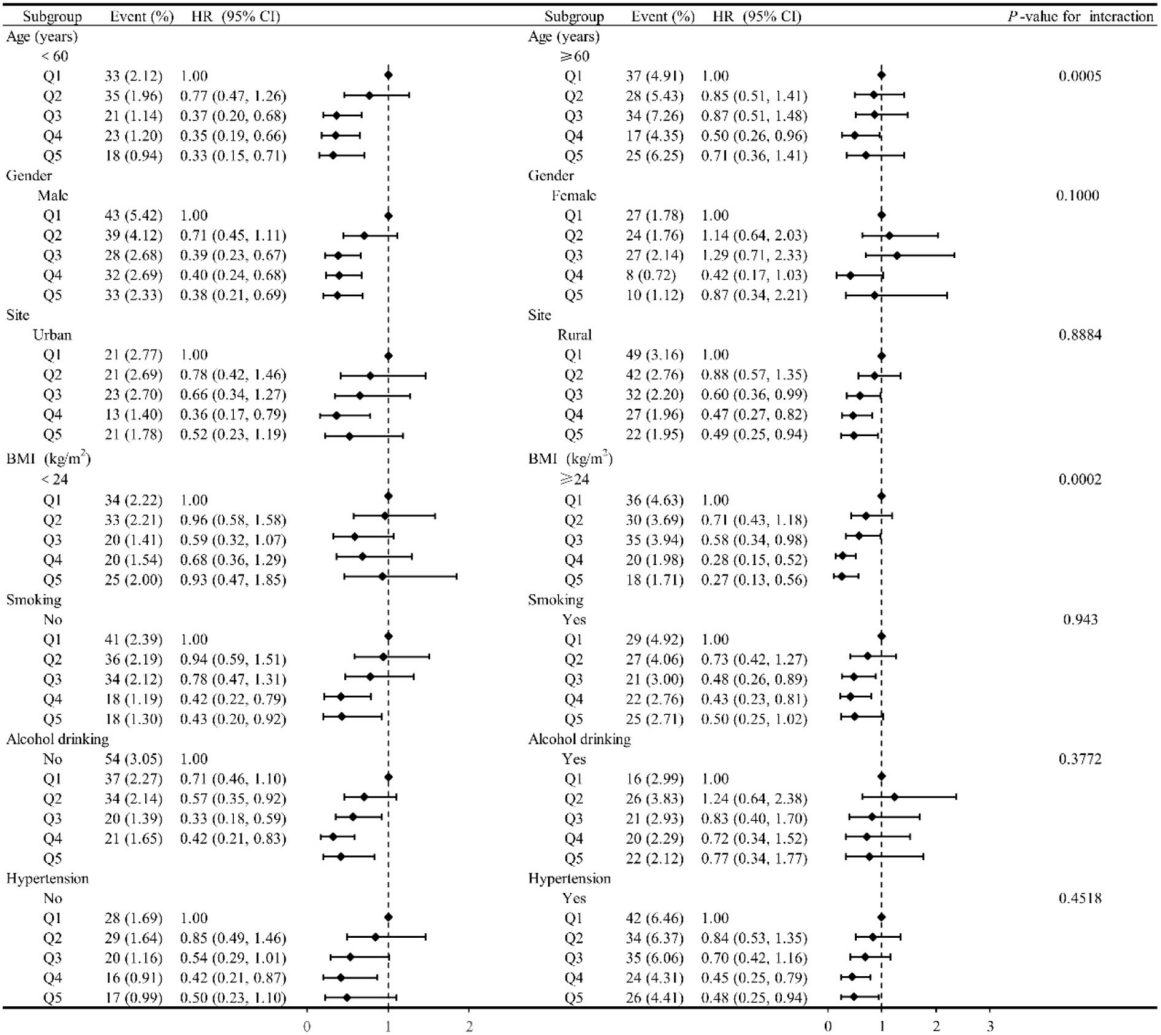
The relationship between dietary selenium intake and risk of developing stroke according to basic features. Except for the stratification component itself, each stratification factor was adjusted for all other variables (age, gender, race, energy intake, site, marital status, education level, activity level, smoking status, drinking status, BMI, hypertensions, myocardial infarction, diabetes, dietary fiber, niacin, vitamin C, vitamin E, calcium, iron, zinc, magnesium, copper, and manganese).

We further performed a tentative analysis to assess whether there is any other potential factor that might affect the inverse relationship between the intake of the selenium and the newly diagnosed stroke, as shown in Figure 3. The influence trends of dietary selenium intake on stroke remained unchanged among most subgroup by gender, cigarette smoking, alcohol drinking, and hypertension status (all *P*-value for interaction >0.0500). However, the association between selenium consumption and stroke risk became drastically changed by age (*P*-value for interaction = 0.0050). The multivariate-adjusted *HR* of stroke for the highest vs. the lowest quintile of selenium intake was 0.33 (95% *CI*: 0.15–0.71, the trend *P*-value of quintile was 0.0018) among aged <60 years, and 0.71 (95% *CI*: 0.36–1.41, the trend *P*-value of quintile was 0.3070) among aged ≥ 60 years. Likewise, we also observed a statistically significant interaction between dietary selenium consumption and BMI in relation to the danger of stroke (*P*-value for interaction was 0.0002), the inverse association for selenium consumption seemed more potent for participants whose BMI ≥24 kg/m^2^, compared with those whose BMI <24 kg/m^2^.

## 4. Discussion

Our research revealed that increasing dietary selenium intake in the habitual low selenium intake population can significantly reduce the risk of stroke, and this relationship was nonlinear L-shaped. When selenium intake did not exceed the RNI, the risk of stroke was significantly decreased with the increase of selenium intake, and the risk of stroke was reduced by 25% with every increase of 10 μg/day selenium intake. However, there was no further benefit from increasing selenium intake when it exceeded RNI.

Almost all studies from Chinese population have found that selenium has a negative effect on stroke (3, 28, 40–43). However, selenium in these studies was assessed by blood selenium, lack of evidence of dietary selenium and stroke. Serum selenium is widely regarded as an indicator of the long-term selenium status of the organism (44), but its concentration is influenced by many factors (although the influence is small) (45). Considering this situation, other evaluation indicators of selenium are particularly important for the impact of stroke. Our research makes up for this literature gap.

Several studies from other countries are consistent with our results (18, 19, 26, 46). Randomized controlled trial from Iran shows that selenite supplementation can improve short-term prognosis in patients with acute ischemic stroke (46). A study from Inuit in Canada found a negative L-shaped relationship between serum and dietary selenium and stroke, and the estimated turning points of the L-shaped curve for blood selenium and dietary selenium were 450 μg/L and 350 μg/day, respectively (26). However, it is inappropriate to extrapolate the results to the general population due to the high intake of selenium in the above study population, which was predominantly from marine mammals (26). Shi W et al. found that increased dietary selenium intake significantly reduced the risk of stroke in the general US population, with 105 μg/day being the nodal point and no further benefit from increasing selenium intake beyond 105 μg/day (18). These studies have demonstrated the importance of selenium in the prevention of strokes. In this study, the RNI of selenium was used as the node of significance, before which the risk of stroke decreased significantly with increasing selenium intake, whereas, the effect of dietary selenium on stroke was not significant after the node. In conjunction with previous studies that have shown narrow safety limits for selenium (30), We recommended that dietary selenium intake should be increased to 50–60 μg/day in the general population of China to maximize stroke prevention and maintain health, which emphasizes the importance of RNI and EAR of selenium.

Stroke is one of the most common cardiovascular diseases and there are no uniform conclusions regarding the relationship between selenium and cardiovascular disease. In Italians aged >60 years, subjects with plasma selenium <60 μg/L were 1.9 times more likely to develop cardiovascular disease than subjects with adequate plasma selenium (47). Randomized controlled trial of pregnant women shows that selenium supplementation is significantly associated with reduced incidence of pregnancy-induced hypertension (48). Selenium deficiency is associated with the adverse development of vascular dysfunction in patients at high risk of cardiovascular events (49). Two cohort studies from China also found potential benefits of selenium on cardiovascular disease, with dietary selenium intake negatively associated with all-cause mortality and cardiovascular mortality, with a 34% and 21% lower risk of cardiovascular disease mortality in men and women in the highest quintile of selenium intake, respectively, compared to the lowest quintile (50). The selenium intake of this study population (51.34 μg/day for men and 45.48 μg/day for women) was similar to our study.

However, GWAS data based on European populations showed no causal relationship between selenium and ischemic stroke and its subtypes (51). Several randomized controlled trials have similarly shown no effect of selenium supplementation on CVD (52, 53). However, the recruited trial subjects were either patients with CVD risk factors (52) or the US population with adequate selenium intake (selenium intake >130 μg/day and plasma selenium >140 μg/L) (53). The results of their study may not be suitable for the Chinese general health population with habitually low selenium intake. In contrast, the results of our study are more general. Studies have shown that serum/plasma selenium concentrations of 100–120 μg/L are sufficient to optimize the function of glutathione peroxidase (GPx) and selenoprotein P (19). Selenium peroxidase and selenoprotein P function may already be at optimal levels in the populations included in the above trials, and it seems reasonable that selenium supplementation did not further reduce CVD risk. In our study, increasing selenium intake when greater than the RNI for selenium also did not result in a greater benefit for stroke. Therefore, baseline selenium levels should also be taken into account when analyzing the relationship between selenium and stroke (28).

High levels of selenium are toxic (54). The Food and Nutrition Board (FNB) at the Institute of Medicine of the National Academies, US, recommends an EAR of 45 μg/day for selenium, a recommended dietary allowance (RDA) of 55 μg/day for selenium, and a tolerable upper intake limit (UL) of 400 μg/day for selenium for men and women aged 19–50 years (55). The recommended reference nutrient intake (RNI) of Se for adults in the United Kingdom (UK) is 60 μg/day for adult women and 75 μg/day for lactating women and adult men (33). The EAR, RNI and UL of selenium for Chinese adults are 50, 60 and 400 μg/day, respectively (39). Some studies show a U-shaped correlation between selenium and CVD and other health outcomes (11, 14, 56). No toxic effects of selenium were found in this study. A possible explanation is that daily intake of selenium below 55 μg is accompanied by low expression of selenoproteins (57). The average intake of selenium in this study was 47.36 μg/day, which may not be sufficient for optimal expression of selenoproteins, let alone the potential toxicity of high levels of selenium. In addition, subgroup analysis suggested that the association between selenium and stroke was stronger in the group with high selenium intake. Similar findings have been reported in previous studies (18, 28, 58).

Although the underlying mechanism for the negative association of selenium with stroke is not known, the relationship is biologically plausible. An important physiopathological mechanism in the development of stroke is oxidative stress, and selenium attenuates cerebral ischaemia/reperfusion injury by regulating oxidative stress, mitochondrial fusion and iron death (5, 59, 60). Secondly, elevated levels of inflammatory markers such as neutrophil/lymphocyte ratio, serum CRP levels and copper/zinc ratio in selenium-deficient patients, in addition to enhanced gene expression of some cytokines and chemokines found in PBMCs of selenium-deficient CVD patients, suggest that reduced selenium levels may favor the progression of inflammation and promote the development of CVD (61). Besides, selenium supplementation may improve the antioxidant capacity of patients with coronary artery disease (CAD) by increasing GPx-1 activity (62). Finally, selenium may act as an antidote to some heavy metals (e.g., mercury, arsenic) and prevent their harmful effects on cardiovascular disease (27, 63).

Most people are exposed to selenium almost exclusively through their diet (13). Brazil nuts, seafood and organ meats are the richest food sources of selenium (64). Studies have shown that selenium-rich Brazil nuts can improve oxidative stress by regulating GPx activity and the expression of Nrf2-related genes (65). In addition, selenium is a component of dietary antioxidants and increasing dietary antioxidant intake may not only reduce the risk of stroke but also improve the prognosis of stroke patients (66, 67). Although many studies have demonstrated the beneficial effects of selenium on stroke, a balanced diet (containing fruits, vegetables, legumes, whole grain cereals, nuts and seeds) naturally rich in antioxidant foods such as selenium may be a safer approach until the mechanistic effects of selenium and antioxidant supplement use on stroke are elucidated (68).

## 5. Strengths and limitations of this study

Strengths: This is a cohort study that can be used to explore the causal relationship between selenium and stroke. Secondly, our research on dietary selenium and new-onset stroke in the population with low selenium intake filled this literature gap. Limitations: First, stroke outcomes are self-reported, which may be influenced by recall bias. However, there is evidence to support the validity of using self-report methods (69). Secondly, there is no information on stroke subtypes, which complicates speculation on potential mechanisms for the beneficial effects of selenium on stroke. Thirdly, the unavailability of selenium supplements may lead to an underestimation of selenium, however, studies have shown that the use of nutritional supplements in China is extremely low. Fourthly, there are differences in the bioavailability of selenium from different foods and there is a need to conduct studies on the effects of dietary selenium intake from different sources on stroke.

## 6. Conclusion

In conclusion, in the general population with habitually low selenium intake, there was a non-linear L-shaped association between dietary selenium intake and risk of stroke incidence. That is, increasing dietary selenium intake significantly reduced the risk of stroke in the presence of inadequate selenium intake, and the beneficial effect of selenium on stroke became insignificant in the presence of adequate selenium. In addition, given the narrow safety range of selenium and the fact that the mechanism of action of selenium in stroke is not yet clear, it is recommended to limit dietary selenium intake to a certain range by increasing selenium-rich foods to minimize strokes and avoid potential adverse health effects of high selenium intake.

## Data availability statement

The datasets presented in this study can be found in online repositories. The names of the repository/repositories and accession number(s) can be found below: https://www.cpc.unc.edu/projects/china.

## Ethics statement

The studies involving human participants were reviewed and approved by the Institutional Review Boards of the National Institute of Nutrition and Food Safety of China (Beijing) and the University of North Carolina (Chapel Hill, NC, USA). The patients/participants provided their written informed consent to participate in this study.

## Author contributions

HZ: formal analysis, methodology, visualization, and writing—original draft. HQ: conceptualization and funding acquisition. YZ: funding acquisition, supervision, and validation. SW: writing—review and editing, supervision, and validation. All authors contributed to the article and approved the submitted version.
