# Supplementary material for: Association of habitually low intake of dietary selenium with new-onset stroke: A retrospective cohort study (2004–2015 China Health and Nutrition Survey)
Source: Front Public Health. 2023 Jan 9;10:1115908. doi: 10.3389/fpubh.2022.1115908 (PMC9869146; doi:10.3389/fpubh.2022.1115908)
Supplement: Supplementary file 1 [file Table_1.pdf]

Table S1. Baseline characteristics of participants among non-stroke and new-onset stroke

Characteristics	Total	Non-stroke	New-onset stroke	*P*-value
N	11532	11261	271	
Male, no. (%)	5389 (46.73)	5214 (46.30)	175 (64.58)	<0.0001
Han race, no. (%)	10276 (89.11)	10036 (89.12)	240 (88.56)	0.7696
Urban, no. (%)	4502 (39.04)	4403 (39.10)	99 (36.53)	0.3918
Smoking, no. (%)	3673 (31.85)	3549 (31.52)	124 (45.76)	<0.0001
Drinking, no. (%)	3838 (33.28)	3733 (33.15)	105 (38.75)	0.0534
Hypertension, no. (%)	2909 (25.23)	2748 (24.40)	161 (59.41)	<0.0001
Diabetes, no. (%)	264 (2.29)	245 (2.18)	19 (7.01)	<0.0001
Myocardial infarction, no. (%)*	48 (0.42)	39 (0.35)	9 (3.32)	<0.0001*
Marital status, no. (%)				
Never married	688 (5.97)	685 (6.08)	3 (1.11)	<0.0001
Married	9940 (86.19)	9708 (86.21)	232 (85.61)	
Divorced, separated, widowed, etc	904 (7.84)	868 (7.71)	36 (13.28)	
Education level, no. (%)				
≤ Primary school	4399 (38.15)	4238 (37.63)	161 (59.41)	<0.0001
Middle school	3736 (32.40)	3674 (32.63)	62 (22.88)	
≥ High school	3397 (29.46)	3349 (29.74)	48 (17.71)	
Activity level, no. (%)				
Light	6201 (53.77)	6050 (53.73)	151 (55.72)	0.0007
Moderate	1752 (15.19)	1732 (15.38)	20 (7.38)	
Heavy	3579 (31.04)	3479 (30.89)	100 (36.90)	
Age (years)	48.00 (37.00, 58.00)	48.00 (37.00, 58.00)	60.00 (53.00, 68.00)	<0.0001
BMI (kg/m^2)	23.03 (20.90, 25.46)	23.01 (20.88, 25.42)	24.14 (21.71, 26.98)	<0.0001
Energy (kcal/day)	1771.96 (1440.18, 2129.10)	1772.40 (1440.21, 2129.18)	1743.84 (1437.57, 2108.57)	0.9714
Dietary fiber (g/day)	11.08 (8.33, 14.74)	11.07 (8.33, 14.72)	11.65 (8.49, 16.01)	0.1908
Niacin (mg/day)	14.31 (10.96, 18.14)	14.33 (10.97, 18.15)	13.3 (10.26, 17.58)	0.0418
Vitamin C (mg/day)	85.00 (59.47, 114.80)	84.98 (59.5, 114.87)	85.83 (58.67, 113.10)	0.8948
Vitamin E (mg/day)	10.66 (7.43, 15.00)	10.65 (7.42, 14.95)	11.24 (7.77, 16.21)	0.0716
Calcium (mg/day)	371.14 (282.65, 492.35)	371.58 (282.86, 492.53)	352.33 (278.02, 476.17)	0.1345
Iron (mg/day)	18.88 (15.06, 23.65)	18.90 (15.07, 23.68)	18.09 (14.94, 22.95)	0.3556
Zinc (mg/day)	10.48 (8.35, 12.85)	10.49 (8.35, 12.86)	9.87 (8.01, 12.71)	0.0850
Magnesium (mg/day)	279.92 (226.57, 343.59)	279.76 (226.44, 343.48)	284.67 (230.49, 348.32)	0.3614
selenium (μg/day)	42.76 (32.10, 56.46)	42.87 (32.18, 56.59)	39.00 (29.35, 51.31)	0.0013

* Fisher's precision probability test. Median (q1, q3) for continuous variables and numbers (percentage) for categorical variables. BMI: body mass index.

Table S2. Analysis of covariance

Variables	Types of variables	TOL	VIF
Gender	Categorical variables	0.47	2.13
Nationality	Categorical variables	0.93	1.08
Site	Categorical variables	0.77	1.29
Smoking status	Categorical variables	0.56	1.79
Alcohol drinking	Categorical variables	0.67	1.48
Hypertension	Categorical variables	0.82	1.22
Diabetes	Categorical variables	0.96	1.04
Myocardial infarction	Categorical variables	0.98	1.02
Marital status	Categorical variables	0.79	1.26
Education level	Categorical variables	0.67	1.49
Activity level	Categorical variables	0.69	1.45
Age	Continuous variables	0.59	1.71
BMI	Continuous variables	0.91	1.10
Energy	Continuous variables	0.25	4.02
Dietary fiber	Continuous variables	0.39	2.54
Niacin	Continuous variables	0.33	3.02
Vitamin C	Continuous variables	0.70	1.43
Vitamin E	Continuous variables	0.55	1.82
Calcium	Continuous variables	0.32	3.13
Iron	Continuous variables	0.31	3.24
Zinc	Continuous variables	0.16	6.24
Magnesium	Continuous variables	0.17	5.85
Copper	Continuous variables	0.61	1.64
Manganese	Continuous variables	0.55	1.83
selenium	Continuous variables	0.49	2.04

TOL: tolerance; VIF: variance inflation factor. The tolerance of all variables is greater than 0.1 and the variance inflation factor is less than 10, so there is no collinearity between the variables. There was collinearity among energy, protein, fat and carbohydrate, so the three macronutrients were not included as independent variables.

Table S3. Sensitivity analysis of dietary selenium grouped by RNI and EAR.

	Dietary selenium intake (μg/day)			
Total population	< 50	50–60	> 60	*P*-value
Cases	199	29	43	-
N	7502	1676	2354	-
Person-years	51415	12057	14706	-
Incidence density	3.87	2.41	2.92	-
Incidence rate (%)	2.65	1.73	1.83	-
Model 1 *HR* (95%*CI*) [a]	1.00	0.62 (0.42, 0.92)	0.76 (0.55, 1.06)	0.0247
Model 2 *HR* (95%*CI*) [a]	1.00	0.65 (0.43, 0.97)	0.74 (0.51, 1.06)	0.0552
Model 3 *HR* (95%*CI*) [a]	1.00	0.60 (0.40, 0.89)	0.70 (0.48, 1.03)	0.0207
Model 4 *HR* (95%*CI*) [a]	1.00	0.58 (0.39, 0.87)	0.70 (0.48, 1.04)	0.0181
Model 1 *HR* (95%*CI*) [b]	1.61 (1.09, 2.38)	1.00	1.22 (0.76, 1.96)	0.0247
Model 2 *HR* (95%*CI*) [b]	1.54 (1.03, 2.30)	1.00	1.13 (0.70, 1.82)	0.0552
Model 3 *HR* (95%*CI*) [b]	1.68 (1.12, 2.52)	1.00	1.18 (0.73, 1.91)	0.0207
Model 4 *HR* (95%*CI*) [b]	1.73 (1.15, 2.60)	1.00	1.22 (0.75, 1.98)	0.0181

RNI: Recommended Nutrient Intake (60 μg/day for aged 18 years and above); EAR: Estimated Average Requirement (50 μg/day for aged 18 years and above). Incidence density: 1/1000 person-years, *HR*: hazard ratio, *CI*: confidence intervals. [a] the lowest quintile (Q1) was regarded as the reference, [b] the fourth quintile (Q4) was regarded as the reference. **Model 1:** non-adjusted. **Model 2:** adjusted for age, gender, race, and energy. **Model 3:** adjusted for age, gender, race, energy, site, marital status, education level, activity level, smoking status, drinking status, BMI, hypertension, diabetes, and myocardial infarction. **Model 4:** adjusted for age, gender, race, energy intake, site, marital status, education level, activity level, smoking status, drinking status, BMI, hypertension, diabetes, myocardial infarction, dietary fiber, niacin, vitamin C, vitamin E, calcium, iron, zinc, magnesium, copper, and manganese

Table S4. Sensitivity analysis of missing values was performed after multiple imputation.

Total population	Dietary selenium intake (μg/day)					*P*-value
	Q1 (≤ 29.72)	Q2 (29.72–38.50)	Q3 (38.50–47.18)	Q4 (47.18–60.22)	Q5 (≥ 60.22)	
Cases	74	65	57	45	44	-
N	2400	2400	2395	2400	2398	-
Person-years	13917	17188	17779	17350	15059	-
Incidence density	5.32	3.78	3.21	2.59	2.92	-
Incidence rate (%)	3.08	2.71	2.38	1.88	1.83	-
Model 1 *HR* (95%*CI*) [a]	1.00	0.71(0.51, 0.99)	0.60(0.43, 0.85)	0.49(0.34, 0.71)	0.55(0.38, 0.80)	0.0008
Model 2 *HR* (95%*CI*) [a]	1.00	0.82(0.58, 1.16)	0.66(0.45, 0.95)	0.53(0.35, 0.79)	0.52(0.33, 0.81)	0.0128
Model 3 *HR* (95%*CI*) [a]	1.00	0.79(0.56, 1.11)	0.58(0.39, 0.84)	0.44(0.29, 0.68)	0.42(0.26, 0.69)	0.0010
Model 4 *HR* (95%*CI*) [a]	1.00	0.81(0.58, 1.15)	0.60(0.41, 0.87)	0.44(0.29, 0.68)	0.43(0.26, 0.71)	0.0012
Model 1 *HR* (95%*CI*) [b]	2.06(1.42, 2.98)	1.46(1.00, 2.14)	1.24(0.84, 1.83)	1.00	1.14(0.75, 1.72)	0.0008
Model 2 *HR* (95%*CI*) [b]	1.91(1.26, 2.87)	1.56(1.05, 2.32)	1.25(0.84, 1.86)	1.00	0.99(0.65, 1.51)	0.0128
Model 3 *HR* (95%*CI*) [b]	2.26(1.48, 3.45)	1.78(1.19, 2.65)	1.30(0.88, 1.93)	1.00	0.96(0.62, 1.47)	0.0010
Model 4 *HR* (95%*CI*) [b]	2.27(1.48, 3.48)	1.85(1.24, 2.76)	1.35(0.91, 2.01)	1.00	0.98(0.64, 1.53)	0.0012

Incidence density: 1/1000 person-years, *HR*: hazard ratio, *CI*: confidence intervals. [a] the lowest quintile (Q1) was regarded as the reference, [b] the fourth quintile (Q4) was regarded as the reference. **Model 1:** non-adjusted. **Model 2:** adjusted for age, gender, race, and energy. **Model 3:** adjusted for age, gender, race, energy, site, marital status, education level, activity level, smoking status, drinking status, BMI, hypertension, diabetes, and myocardial infarction. **Model 4:** adjusted for age, gender, race, energy intake, site, marital status, education level, activity level, smoking status, drinking status, BMI, hypertension, diabetes, myocardial infarction, dietary fiber, niacin, vitamin C, vitamin E, calcium, iron, zinc, magnesium, copper, and manganese
